# Atheroprotective Mechanisms of Tilianin by Inhibiting Inflammation Through Down-Regulating NF-κB Pathway and Foam Cells Formation

**DOI:** 10.3389/fphys.2019.00825

**Published:** 2019-07-02

**Authors:** Wanli Shen, Gulinigaer Anwaier, Yini Cao, Guan Lian, Cong Chen, Shu Liu, Nuerbiye Tuerdi, Rong Qi

**Affiliations:** ^1^School of Basic Medical Science, Shihezi University, Shihezi, China; ^2^School of Pharmacy, Shihezi University, Shihezi, China; ^3^Institute of Cardiovascular Sciences, Peking University Health Science Center, Peking University, Beijing, China; ^4^Key Laboratory of Molecular Cardiovascular Sciences, Ministry of Education, Beijing, China; ^5^Beijing Key Laboratory of Molecular Pharmaceutics and New Drug Delivery Systems, Beijing, China

**Keywords:** atherosclerosis, foam cell, inflammation, NF-κB pathway, tilianin

## Abstract

Tilianin, a representative flavonoid ingredient of *Dracocephalum moldavica* L., has been used to treat several diseases for centuries, including atherosclerosis (AS). However, pharmacological mechanisms underlying its biological functions remain elusive. In the present study, we investigated the anti-AS mechanisms of tilianin through establishing *in vitro* models using three types of cells that contributed to AS progression, including macrophage, vascular smooth muscle cells and human umbilical vein endothelial cells, which were proved to be involve in LPS/TNF-α/oxidized low density lipoprotein (ox-LDL)-induced inflammation and ox-LDL induced foam cell formation. Our results indicate that tilianin significantly suppressed LPS induced inflammatory responses on macrophage and remarkably inhibited TNF-α induced VSMCs proliferation and migration. Furthermore, the anti-inflammatory effect of tilianin on macrophages and VSMCs was proved to be mainly by downregulating TNF-α/NF-κB pathway. Moreover, our results demonstrate that tilianin significantly ameliorated ox-LDL induced macrophages oriented foam cells formation through repressing mRNA expression of SR-A1 and inducting the expression of genes related to cholesterol efflux including SRB-1 and ABCA1. However, tilianin had no effect on ox-LDL induced HUVECs injury.

## Graphical Abstract

Tilianin inhibits inflammatory responses in macrophage and VSMCs by regulating TNF-α/NF-κB pathway as well as inhibits ox-LDL induced foam cell formation to protect against atherosclerosis.

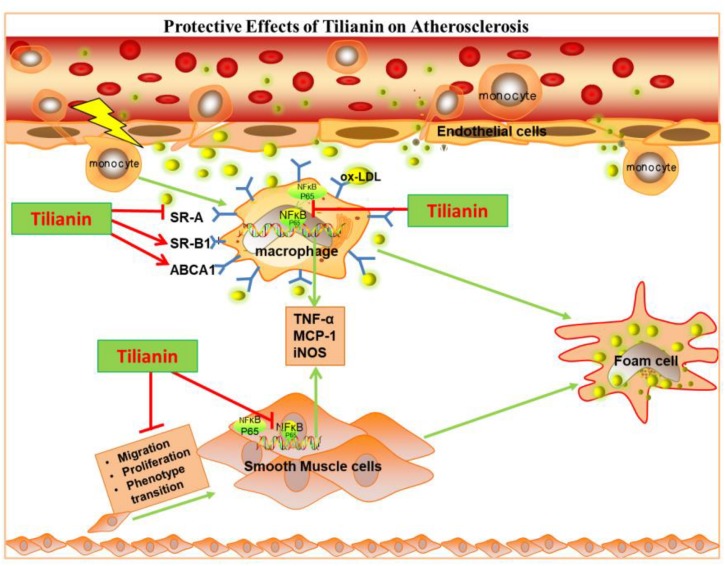

## Introduction

Atherosclerosis (AS), a common cardiovascular disease, has threatened the life of human beings for decades (Loke et al., 2010). With the rapid development of medical technology, researches on AS have sprung up vigorously (Libby et al., 2010). Numerous studies on its pathogenesis have illustrated that AS is in fact a chronic inflammatory disease (Hansson, 2005; Hermansson and Hermansson, 2011) closely associated with endothelial injury (Mano et al., 1996; Korshunov et al., 2007; Park and Park, 2015), inflammatory infiltration and foam cell formation (Korshunov et al., 2007). Endothelial cells (ECs) injury and dysfunction is an initial step for AS (Dash, 2017). Injured ECs secrete various pro-inflammatory molecules, including VCAM-1, ICAM-1, which furthermore recruit monocytes from plasma into early atherosclerotic lesions (Libby, 2002; Tziomalos et al., 2010; Ding et al., 2017). Additionally, pro-inflammatory factors secreted by injured ECs within the plaque lesion will thereby stimulate differentiation of monocytes into macrophages, which phagocytize ox-LDL *via* its surface scavenger receptors to form into lipid-load foam cells (Herschman, 2003; Hegde et al., 2017). Various growth factors and chemokines in plaque area also promote the migration and proliferation of VSMCs, which are at the core of AS diseases. Although initially only an athermanous plaque formed in the vessel wall, eventually the plaque will evolve into an unstable one and ruptures, which along with luminal thrombosis are responsible for most of the acute coronary diseases (Sakakura et al., 2013). To date, anti-inflammation or lipid-lowering drugs have been used for alleviating the morbidity and mortality of atherosclerotic cardiovascular diseases. However, these drugs have limited symptomatic effects and do not delay AS progression, highlighting the unmet need for novel prophylactic and therapeutic agents for AS (Hegde et al., 2017).

Due to their health-promoting properties and low toxicity, herbal medicine has long been used to treat various diseases (Zhang et al., 2016). *Dracocephalum moldavica* L., whose prescription was named as Yixin Badiranjibuya Granules, is one of the most popular herbs in Uyghur medicine and is used as a single medicine prescription in the Uyghur Medicine Ministerial Standard, and has been used to treat a variety of cardiovascular diseases (CVDs) (Feng and Li, 2003; Song et al., 2010). Tilianin is the main flavonoids compound extracted from *D. moldavica* L. (Nam et al., 2006), which has been reported to have anti-AS (Nam et al., 2005), anti-inflammation (Nam et al., 2006), and sedative (Martínez-Vázquez et al., 2011) effects. In addition, tilianin shows protective effects against myocardial ischemia-reperfusion injury of rats (Singh et al., 2010). And tilianin can significantly increase the serum protein level of SOD/GSH-PX, while decreasing the mRNA expression level of *TNF-α* (Nam et al., 2005, 2006). Although tilianin has been reported to have protective effects on AS and was widely used to treat AS in clinic, its anti-AS mechanism has remained unclear so far.

In the present study, we investigated the anti-AS mechanism of tilianin using three types of primary cells, including macrophages, HUVECs and VSMCs, to establish *in vitro* AS models, which were represents inflammation model and form cell model. Our results reveal that the anti-AS mechanisms of tilianin were mainly owing to its inhibitory effect on inflammation in macrophages and VSMCs by down-regulating TNF-α/NF-κB pathway, and attenuation of foam cell formation by regulating mRNA expression of the genes related to cholesterol influx and efflux in macrophages.

## Materials and Methods

### Material

Tilianin was purchased from Cheng Du Angsaisi Bio-Technology Co., Ltd. (HPLC >98%, Supplementary Figure 1) and its concentration was determined by quantifying peak area (Supplementary Table 1). TNF-α was obtained from PeproTech (Rocky Hill, CT, United States). Fetal Bovine Serum (FBS), Dulbecco’s Modified Eagle’s Medium (DMEM) and Roswell Park Memorial Institute 1640 (RPMI) were obtained from GIBCO (Grand Island, NE, United States). BAY 11-7082 was purchased from Beyotime Biotechnology (Jiangsu, China). Goat anti-NF-κB p65 and rabbit anti-α-SMA were purchased from Santa Cruz Biotechnology (CA, United States). Rabbit anti-MMP-2/9 primary antibodies were purchased from Abcam (MA, United States). Rabbit anti-histone primary antibody was purchased from Cell Signaling (MA, United States). Mouse anti-GAPDH antibody was purchased from Santa Cruz (Dallas, TX, United States).

This study was carried out in accordance with the principles of the Basel Declaration and Recommendations of Animal Care and Use Committee of Peking University, PU Laboratory Animal Welfare Committee. The protocol was approved by the PU Laboratory Animal Welfare Committee (Approval No: LA2017193).

### Isolation and Culture of Primary Macrophages

C57BL/6 mice weighting about 20 g were intraperitoneally injected with Broth. Three days after injection, peritoneal lavage was performed in the mice using 10 mL of 0.01 M phosphate-buffer saline (PBS). Individual mice peritoneal cell lavage was treated separately. The cells at a concentration of 2 × 10^6^ cells/mL were pelleted and resuspended in RPMI 1640 cell culture medium with 10% FBS and 100 g/mL streptomycin and 100 IU/mL penicillin. Macrophages were separated and planted in culture plates at 37°C (5% CO_2_) for 4 h according to the literature (Pineda-Torra et al., 2015).

### Isolation and Culture of Primary VSMCs

Primary VSMCs were obtained from thoracic arteries of rats as described in the previous literature (Wang et al., 2015, 2017). Briefly, male SD rats weighing about 100 g were anaesthetized and thoracic arteries were carefully excised, and then the fat tissues, adventitia and other connective tissues around the arteries were separated by blunt dissection. Then the arteries were washed using 0.01 M PBS containing 100 g/mL streptomycin and 100 IU/mL penicillin. Arterial ectoderm was removed and sliced with an ophthalmic scissor, and the vascular endothelium was scratched gently using curved dissection forceps. Then the vascular tissues were washed and cut into small pieces of approximately 1 mm^3^ per size. Then these slices were placed at the bottom of a 100 mm culture dish where 1 mL DMEM with 10% FBS, 100 g/mL streptomycin and 100 IU/mL penicillin was added. The pieces were incubated at 37°C for 6 h until they all stick to the bottom of the cultured dish. In the following days, the culture medium was added gently to the culture dish every 4 days. After 5–7 days, the VSMCs in the 4–7 generations were used in the next experiments.

### Isolation and Culture of Primary ECs

HUVECs were isolated from umbilical veins of fresh cords from donors with written informed consent (Wall et al., 1978). The umbilical veins were digested by collagenase, and the collected HUVECs were cultured in ECM with 5% FBS, 1% endothelial cell growth supplements and 1% penicillin/streptomycin solution. Then the HUVECs were cultured on 0.1% (w/v) gelatin-coated culture flasks and culture flasks were placed in a humidified incubator at 37°C (5% CO_2_). The HUVECs in the passages of 2–5 were used in the following experiments (Baudin et al., 2007).

### MTT Cell Viability Assay

Cell metabolic activity was analyzed using a MTT assay as per manufacturer’s protocol. Briefly, cells (six replicates per group) at a density of 5 × 10^3^ cell/well were cultured in 96-well plates for 24 h. MTT was then added at a final concentration of 0.5 mg/mL for 4 h. Finally, DMSO was used to dissolve the insoluble formazan product. Absorbance values were then read using a microplate reader at 570 nm. All experiments were repeated at least three times.

### Cell Counting Kit-8 (CCK-8) Assay

CCK-8 assay were used to quantify cell proliferation as per manufacturer’s protocol. Briefly, cells (six replicates per group) at a density of 5 × 10^3^ cell/well were seeded in 96-well plates for 24 h. After cell supernatants were aspirated, cell monolayers were washed using PBS twice times, and began starvation for another 24 h. Subsequently, cell supernatants were aspirated, the cell monolayers were washed again, the experiment was divided into normal control group: DMEM culture medium containing 20% FBS; Experimental group: adding 20% FBS DMEM culture and TNF-α (at concentrations of 0.01, 0.1, 1, 10, and 100 ng/mL), at 37°C (5% CO_2_) incubator for 24 h. Then, cell supernatants were aspirated, cell monolayers were washed using PBS, and 10 μL of CCK-8 solution was added to the plate. Incubate the plate for 1 h in CO_2_ incubator. The absorbance of obtained supernatants was read at 450 nm using a microplate reader. Assay was repeated at least three times.

### Cell Migration Assay

The wound healing assay was used to test migration capability of VSMCs. Briefly, cells were incubated in DMEM supplemented with 10% FBS. After 24 h of growth, cells reach at a density of 70–80% confluence as a monolayer were seed into 24-well tissue culture plate. After cells reach at concentration of 70–80%, cells were incubated in DMEM supplemented with 0.5% FBS for 24 h. Then, without changing the medium, the monolayer were scratched gently with a new 200 μL pipette tip across the center of the well. Cells were gently washed with 1× PBS for three times to remove the detached cells and incubated with DMEM supplemented with 0.5% FBS as well as TNF-α and different concentration of tilianin. for additional 24 h. Cells were washed twice with 1× PBS and stained with 1% crystal violet in 2% ethanol for 30 min. Photos were taken for the stained monolayer on a microscope. Multiple views of each well were documented, and each experimental group was repeated multiple times.

### Total Protein Extractions and Western-Blot Analysis

Cells were washed with ice-cold 0.01 M PBS twice. Then, lysis buffer (AIDLAB) containing 10% protease inhibitor was used and some amount of cell lysates were heated at 95°C for 10 min, while some were used for the quantify of protein concentration with a BCA kit (Pierce Biotechnology, Rockford, IL, United States). Cell lysates containing 25 μg of protein were separated on SDS/PAGE gels and electro-transferred onto a polyvinylidene difluoride membrane using the Bio-Rad Mini-Protean II apparatus (Bio-Rad Laboratories, Carlsbad, CA, United States). After blocking with Tris-buffered saline/0.1% Tween-20 containing 5% fetal bovine serum at room temperature for every 30 min to 1 h, membranes were infiltrated in the corresponding primary antibodies for at least 12 h. After incubating with the specific peroxidase-conjugated secondary antibodies, the membranes were detected with enhanced chemiluminescence system (Pierce Biotechnology, Rockford, IL, United States). GAPDH and Histone was used as an internal control. Bands were quantified using ImageJ software.

### Nuclear Protein Isolation

After different incubation conditions, cells were washed with ice-cold 0.01 M PBS twice and suspended with 2 mL Buffer A (10 mM Tris, 10 mM KCl, 1.5 mM MgCl_2_, 0.5% NP40) and re-suspended with 1 mL Buffer A. After 10 min, nuclei were collected by centrifugal at 4700 *g* (Thermo Electron LED GmbH, Am Kalkberg, 37520 Osterode, Germany) for 5 min at 4°C. Then, the nuclear proteins were extracted with 500 μL of Buffer (20 mM Tris (pH = 7.4), 1.5 mM MgCl_2_, 420 mM NaCl, 10% glycerol). After incubating at 4°C for 30 min, the samples were frozen by liquid nitrogen thrice and centrifuged (Thermo Electron LED GmbH, Am Kalkberg, 37520 Osterode, Germany) at 13800 *g* for 20 min. The nuclear extracts were then quantified for protein concentrations by using the BCA kit (Pierce Biotechnology, Rockford, IL, United States). The nuclear extracts were used immediately for western blot or stored at −80°C.

### RNA Extraction and Quantitative Real-Time PCR

Total RNA from the cells was extracted using TRIzol reagent (Beijing TransGene Biotech Co., Ltd., Beijing, China) and first-strand cDNA was generated by using an RT kit (Abcam, Canada). Primer sequences used for Quantitative RT-PCR analysis were given in Supplementary Table 2. The real-time PCR cycling parameters were set as follows: 95°C for 10 min, followed by 95°C for 30 s, 60°C for 30 s, and 72°C for 30 s for 40 cycles. Finally the mRNA expression levels were indicated with 2^–ΔΔCT^ and normalized to β-actin.

### Oil Red O Staining

The peritoneal macrophages cultured in 12-well palates were pre-incubated for 24 h with ox-LDL (50 μg/mL). The cells were fixed with 4% paraformaldehyde and then washed with 0.01 M PBS and stained with oil red O at 37°C for 30 min and cell morphology was observed.

### Intracellular Cholesterol Measurement

The peritoneal macrophage cultured in 6-well palates was incubated with 50 μg/mL ox-LDL for 24 h. Then cells were washed with 0.01 M PBS twice, intracellular cholesterols were extracted by adding 0.5 mL hexane: isopropanol (3:2) to special vials and dried in fume hood. The Amplex Red Cholesterol Assay Kit (cat. #A12216, Invitrogen) was used to determine the total cholesterol levels in the foam cells, results were normalized using the protein contents detected by the BCA kit.

### Statistical Analysis

The data are presented as means ± standard error of mean (SEM). Multiple group means were compared using one-way ANOVA followed by Bonferroni *post hoc* test. Statistical analysis was performed using Graph Pad Prism 6.0 software package (Graph Pad Software Inc., San Diego, CA, United States). All values were considered statistically significant when *p* < 0.05.

## Results

### Effects of Tilianin on Cell Viability

**FIGURE 1:**
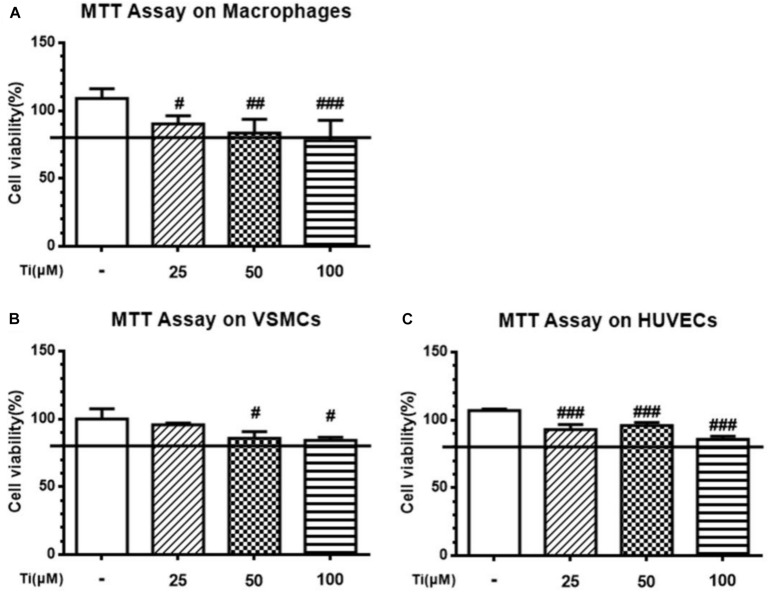
Cytotoxicity of tilianin at different concentrations on the three types of AS-related primary cells. **(A)** Macrophages, **(B)** VSMCs, and **(C)** HUVECs. All data represent the mean ± SEM. ^#^*P* < 0.05, ^##^*P* < 0.01, ^###^*P* < 0.001 vs. control group.

Cells were first incubated with tilianin at different concentrations and cell viability was analyzed using MTT assay. As shown in Figure 1, MTT assay result shows that the cytotoxicity of tilianin elevated with the increase of its concentrations. Maximum safety concentration of tilianin on macrophage, VSMCs and HUVECs was all less than 100 μM. Therefore, 50 μM tilianin as high concentration group and 5 μM tilianin as low concentration group were chosen for the subsequent cell experiments.

### Tilianin Inhibited LPS Induced Inflammation in Macrophages

**FIGURE 2:**
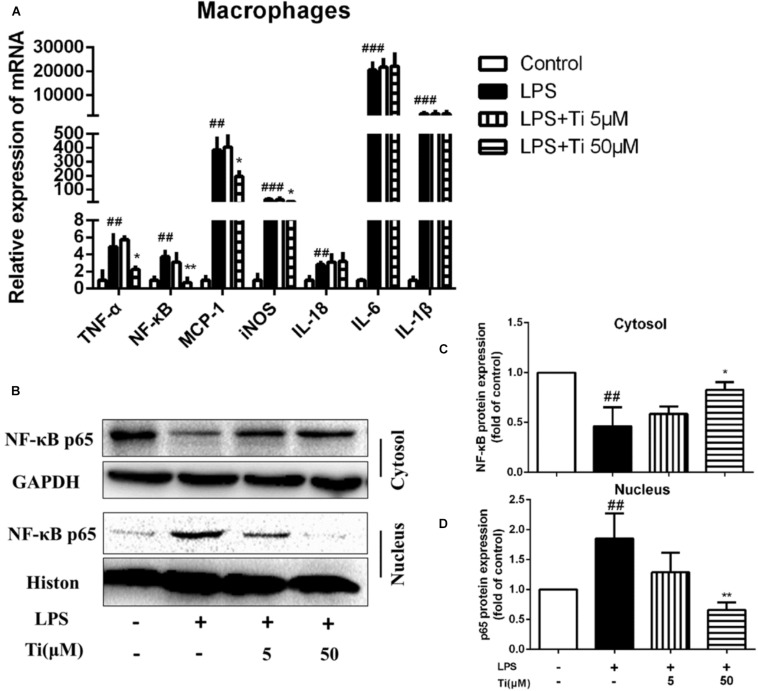
Effects of tilianin on mRNA expression of inflammatory factors and protein level of NF-κB p65 in LPS stimulated macrophages. **(A)** Effect of tilianin on mRNA expression of TNF-α/NF-κB/MCP-1/iNOS/IL-18/IL-6/IL-1β. **(B)** Effect of tilianin on protein expression of NF-κB p65 in macrophages by western blot. **(C,D)** Quantification results of NF-κB p65 expression in cytoplasm **(C)** and nucleus **(D)**. All data represent the mean ± SEM. ^##^*P* < 0.01, ^###^*P* < 0.001 vs. control group, ^*^P < 0.05, ^∗∗^*P* < 0.01 vs. LPS group.

LPS stimulation induced inflammatory responses in macrophages is one of the classical AS inflammatory cell models. Therefore, we co-incubated peritoneal macrophages with 1 ng/mL LPS and tilianin for 12 h to determine expression of inflammatory cytokines. Figure 2A shows that in the LPS group, the mRNA expressions of inflammatory cytokines, including *TNF-α*, *NF-κB*, *MCP-1*, *iNOS*, *IL-1β*, *IL-6*, and *IL-18* were all significantly up-regulated. Nevertheless, incubation of the cells with the high concentration (50 μM) of tilianin significantly decreased mRNA expressions of *TNF-α*, *NF-κB*, *MCP-1*, and *iNOS*, but the low concentration (5 μM) of tilianin showed no significant effect on these inflammatory cytokines. It was reported that tilianin inhibited IκB kinase activation and subsequent phosphorylation, degradation of IκB in LPS-stimulated primary macrophages (Nam et al., 2005). On this basis, we further proved that protein level of nuclear NF-κB p65 was significantly upregulated while cytoplasmic NF-κB p65 was decreased in LPS group (Figure 2B). However, high concentration (50 μM) of tilianin treatment significantly inhibited nuclear translocation of NF-κB p65, and Figures 2C,D are representative quantification results of NF-κB expression in cytosol and nucleus, respectively. These results suggest that tilianin inhibits macrophage inflammatory response through regulating NF-κB signaling pathway. To further prove our hypothesis, we used NF-κB inhibitor BAY 11-7082 to block NF-κB signaling pathway. Results show that BAY 11-7082 successfully inhibited upregulation of phospho-NF-κB. Tilianin inhibitory effects were abolished after BAY 11-7082 treatment (Supplementary Figure 5). Therefore, it is proved that tilianin inhibits LPS induced NF-κB signaling pathway activation to ameliorate inflammatory response of macrophages.

### Tilianin Inhibited TNF-α Induced Inflammation on VSMCs

**FIGURE 3:**
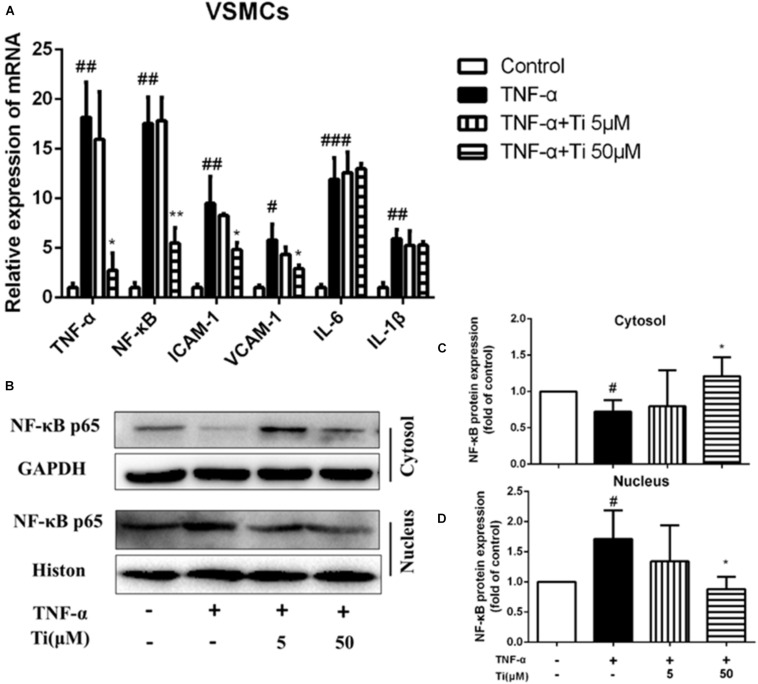
Effects of tilianin on mRNA expression of inflammatory factors and protein expression of NF-κB p65 in TNF-α induced VSMCs. **(A)** Effects of tilianin on mRNA expression of TNF-α/NF-κB/ICAM-1/VCAM-1/IL-6/IL-1β. **(B)** Effects of tilianin on protein expression of NF-κB p65 in VSMCs by western blot. **(C,D)** Quantification results of NF-κB expression in cytoplasm **(C)** and nucleus **(D)**. All data represent the mean ± SEM. ^#^*P* < 0.05, ^##^*P* < 0.01, ^###^*P* < 0.001 vs. control group, ^*^P < 0.05 vs. TNF-α group, ^∗∗^*p* < 0.01.

Then, TNF-α induced inflammation on VSMCs was used as another typical cell model related to AS. Figure 3A shows that the mRNA expression of inflammatory cytokines, including *NF-κB*, *TNF-α*, *ICAM-1*, *VCAM-1*, *IL-1β*, and *IL-6* were significantly up-regulated after incubating VSMCs with 100 ng/mL TNF-α for 24 h, indicating that inflammation model was constructed successfully on VSMCs. NF-κB as a transcriptional factor exists in many cells types. Target genes of NF-κB include immune-related cytokines, adhesion molecules, etc. Our results after treating the cells with high concentration (50 μM) of tilianin, the gene expressions of *TNF-α*, *NF-κB*, *ICAM-1*, and *VCAM-1* were significantly downregulated, but the gene expression levels of *IL-6* and *IL-1β* were not affected obviously. The protein levels of NF-κB in nucleus and cytosol of VSMCs were detected consequently. Interestingly, we found tilianin also can inhibit nuclear translocation of NF-κB in TNF-α-stimulated VSMCs. As is shown in Figure 3B, protein levels of nuclear NF-κB p65 were increased while cytoplasmic NF-κB p65 were decreased in the TNF-α group. And the nuclear translocation of NF-κB p65 induced by TNF-α was significantly inhibited in the high concentration (50 μM) of tilianin group (Figures 3C,D). Besides, both the mRNA expressions and protein levels of inflammation related factors MMP2 and MMP9 were also significantly down-regulated after the high concentration (50 μM) of tilianin treatment (Supplementary Figure 2). But the low concentration (5 μM) of tilianin showed no significant effect on these inflammation related factors.

To sum up, the above experimental results show that, in both macrophages and VSMCs, tilianin significantly inhibited gene expression levels of *TNF-α* and *NF-κB*, but had no obvious effect on gene expression levels of *IL-6* and *IL-1β*, indicating that inhibitory effects of tilianin on inflammation were mainly through down-regulating TNF-α/NF-κB pathways. To further prove our results, we also used NF-κB inhibitor BAY 11-7082 to block NF-κB signaling pathway. Results show that BAY 11-7082 successfully inhibited upregulation of phospho-NF-κB in VSMCs. Tilianin inhibitory effects were abolished after BAY 11-7082 treatment (Supplementary Figure 6). These results proves further that tilianin inhibits TNF-α/NF-κB signaling pathway activation to ameliorate inflammatory response of VSMCs.

### Tilianin Inhibited Migration and Proliferation of VSMCs Induced by TNF-α

**FIGURE 4:**
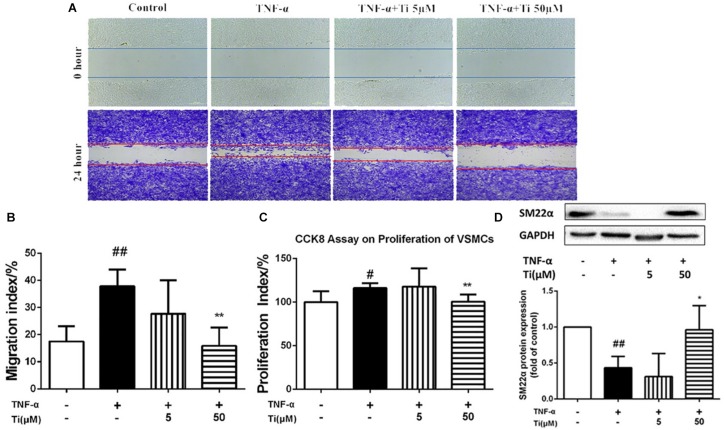
Effects of tilianin on migration and proliferation of VSMCs induced by TNF-α. **(A,B)** Images **(A)** and quantification **(B)** of the migration of VSMCs. **(C)** Proliferation of VSMCs determined by CCK8 kit. **(D)** Protein expression and quantification of SM22α in VSMCs. All data represent the mean ± SEM. ^#^*P* < 0.05, ^##^*P* < 0.01 vs. control group, ^*^P < 0.05, ^∗∗^*P* < 0.01 vs. TNF-α group.

In general, TNF-α stimulation not only activates the inflammatory response as mentioned above, but also promotes proliferation, migration and phenotypic transition of VSMCs, which are critical events in the pathogenesis of AS (Moe and Chen, 2004). Our results displayed in Figure 4A indicate that migration of VSMCs was significantly increased after TNF-α stimulation, and the high concentration (50 μM) of tilianin treatment significantly inhibited such migration of VSMCs, but the low concentration (5 μM) of tilianin has failed to exhibit same effect (Figure 4B). CCK-8 assay also revealed that the high concentration (50 μM) of tilianin effectively inhibited the proliferation of VSMCs (Figure 4C).

Besides TNF-α, ox-LDL was also reported to induce proliferation and synthetic behavior of VSMCs to induce the transition of contractile phenotype of VSMCs toward synthetic phenotype (Zhao et al., 2005). The abnormal proliferation of VSMCs induced by ox-LDL was proved in our present study, and incubation with Tilianin was capable of inhibiting cells proliferation (Supplementary Figure 3).

Moreover, we also measured the protein expression levels of contractile phenotype VSMC marker SM22α in the model group and the tilianin incubation group to determine whether tilianin regulate VDMCs phenotypic modulation. Our results proved that the protein expression of SM22α was significantly down-regulated after by either TNF-α (Figure 4D) or ox-LDL (Supplementary Figure 3) stimulation. And tilianin treatment could significantly inhibit the decrease of SM22α expression induced by either TNF-α or ox-LDL in VSMCs, indicating that the phenotype transformation of VSMCs from the normal contractile phenotype to the inflammatory synthetic phenotype could be suppressed by tilianin, which further explained the inhibitory effects of tilianin on proliferation, migration as well as phenotype switch of VSMCs induced by TNF-α and ox-LDL.

In conclusion, the above results showed that one of the mechanisms of tilianin in amelioration of AS was through inhibition of inflammation.

### Tilianin Inhibited Foam Cell Formation

Macrophages derived foam cell formation is one hallmarks of early AS lesions in both human AS disease and AS animal models. Foam cells are defined as fat-laden M2 macrophages that contain a lot of lipid droplets, which can be stained by Oil red O staining (Mallat, 2014). Aiming to define the potential mechanism by which tilianin reduced lipid content in the macrophages and explain the decrease of foam cells formation, lipid accumulation model by ox-LDL was constructed in macrophages.

**FIGURE 5:**
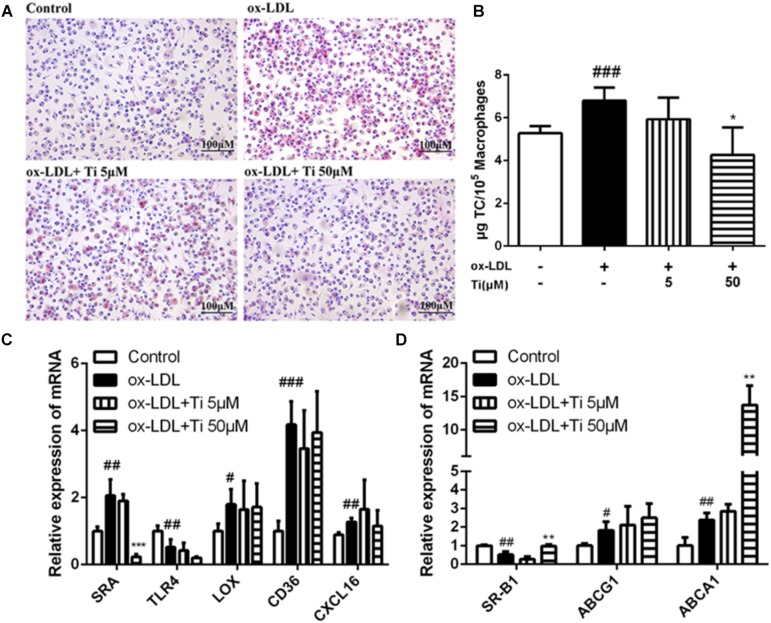
Effects of tilianin on the foam cell formation induced by ox-LDL in macrophages. **(A)** Oil red O staining of the macrophages. **(B)** Quantification of TC content in macrophages. **(C,D)** Effects of tilianin on mRNA expression levels of the genes related to lipid influx **(C)** and efflux **(D)** in macrophages. All data represent the mean ± SEM. ^#^*P* < 0.05, ^##^*P* < 0.01, ^###^*P* < 0.001 vs. control group, ^*^P < 0.05, ^∗∗^*P* < 0.01, ^∗∗∗^*P* < 0.001 vs. TNF-α group.

Lipid accumulation and foam cell formation were induced by ox-LDL stimulation in macrophages and the intracellular lipids were thereby tested by Oil red O staining. Figure 5A indicates that lipid accumulation in the ox-LDL group was much more severe than the normal control group. Nevertheless, compared to the ox-LDL group, the high concentration (50 μM) of tilianin treatment significantly decreased the lipid accumulation in macrophages, and the number of foam cells was also significantly reduced in the high concentration (50 μM) of tilianin group. The results of lipid extraction in macrophages revealed that the cholesterol levels of ox-LDL group were significantly higher than normal control group. And such increase of lipid contents by ox-LDL was significantly reduced after treatment with the high concentration (50 μM) of tilianin, however, the low concentration (5 μM) of tilianin showed no significant effect (Figure 5B).

Generally, the accumulation of large amounts of lipid droplets in ox-LDL derived macrophages foam cell resulted from the increase of cholesterol uptake into the cells and the decrease of cholesterol efflux from the cells. Macrophage scavenger receptors (SRs) including SRA and CD36 mediate internalization of lipoproteins. Cholesterol efflux, which is mostly mediated by ATP-binding cassette transporters ABCA1, ABCG1 as well as by another SRs, like SR-B1 for reverse cholesterol transport, also contributed to foam cell formation (Yvan-Charvet et al., 2010). Therefore, in our study, the mRNA expressions of the genes related to lipid influx (*SRA*, *LOX*, *CD36*, *TLR4*, and *CXCL16*) and efflux (*SR-B1*, *ABCG1*, and *ABCA1*) were detected, and the results showed that mRNA expression level of *SRA* was significantly down-regulated (Figure 5C) while the mRNA expressions of *SR-B1* and *ABCA1* were significantly up-regulated after treatment with the high concentration (50 μM) of Tilianin (Figure 5D), but the mRNA levels of other genes were not significantly affected by tilianin.

Taken together, tilianin inhibited the expression of *SR-A1* to reduce the uptake of cholesterol. What’s more, tilianin elevated the expression of cholesterol efflux genes like *SRB-1* and *ABCA1* to promote cholesterol efflux. Therefore, all of these results provide compelling evidence that another possible mechanism of tilianin on the amelioration effect on the formation AS plaques was through the inhibition of foam cell formations.

### Tilianin Had No Protective Effects on Endothelial Cell Injury

**FIGURE 6:**
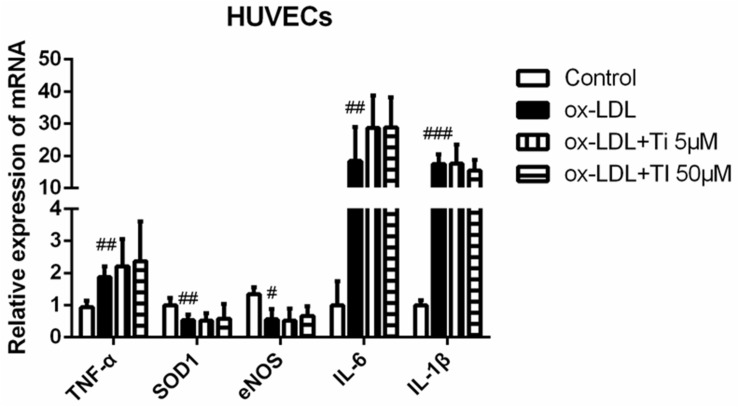
Effects of tilianin on the mRNA expressions of the genes related to the inflammation and oxidative stress induced by ox-LDL in HUVECs. All data represent the mean ± SEM. ^#^*P* < 0.05, ^##^*P* < 0.01, ^###^*P* < 0.001 vs. control group.

Oxidized low density lipoprotein can damage endothelial cells and VSMCs by enhancing the inflammation of blood vessels (Wiesner et al., 2010; Maskrey et al., 2011). Gene expressions of the cytokines related to endothelial cell injury were detected. Figure 6 shows that the mRNA expression of *TNF-α*, *IL-6*, and *IL-1β* were significantly elevated and the mRNA expression of *SOD-1* and *eNOS* were significantly decreased after stimulation of HUVECs with ox-LDL, indicating that endothelial cell injury model was successfully established. However, tilianin treatment exhibited no effective influence on the mRNA expression of the genes mentioned above. Nevertheless, there is still insufficient direct evidence for potential reversion effect of tilianin on endothelial cell dysfunction, so it needs to be completely elucidated in our future study.

## Discussion

Inflammatory infiltration and hyperplasia are the common pathological manifestation of many inflammatory diseases, including AS, which was used to be considered only as a lipid disorder disease, but actually involves persistent inflammatory reactions. Recent advances in basic medical sciences have established the fundamental role of inflammation in all stages of AS, from initiation to progression, and ultimately to the formation of thrombotic complications.

Our data demonstrate that tilianin has significant inhibitory effects on chronic inflammatory responses involved in AS procession, and its anti-inflammatory effects were found in both LPS induced inflammation in macrophages and TNF-α or ox-LDL induced inflammation in VSMCs.

Firstly, LPS was used to induce inflammatory responses on macrophages. LPS is a component of the cell wall of gram-negative bacteria. When macrophages at resting state comes across these stimulus, intense inflammation process will be started, including release of pro-inflammatory cytokines, such as IL-1β, IL-12, TNF-α, and MCP-1, which induce iNOS release so as to attract more mononuclear cells.

Secondly, TNF-α was used to induce inflammation in VSMCs. TNF-α is a pro-inflammatory cytokine that possess many significant pathophysiological functions like inducing cell necrosis and apoptosis as well as affecting embryonic development. TNF-α can induce the generation of other pro-inflammatory cytokines and chemokines, and increase its own production *via* autocrine (Balkwill, 2009).

In both macrophages and VSMCs, tilianin significantly inhibited gene expression of *TNF-α* and *NF-κB*, but had no obvious effect on gene expression of *IL-6* and *IL-1β*, indicating that inhibitory effects of tilianin on inflammation were mainly through down-regulating TNF-α/NF-κB pathways. It was reported that tilianin could regulate nuclear translocation of NF-κB (Nam et al., 2005). And our results in Figures 3B,D further proved that tilianin could inhibit nuclear translocation of NF-κB induced by either LPS or TNF-α. To further prove our hypothesis, we also used NF-κB inhibitor BAY 11-7082 to block NF-κB signaling pathway. Results show that BAY 11-7082 successfully inhibited activation of NF-κB signaling, while the inhibitory effects of Tilianin were abolished after treatment of the macrophages or VSMCs with BAY 11-7082 (Supplementary Figures 5, 6). All of the above results demonstrate that tilianin inhibits NF-κB signaling pathway activation to ameliorate inflammatory response of the cells involved in AS procession.

NF-κB that exists in many types of cells, is one of the most vital transcription factors, whose target gene includes immune-related cytokines, receptors, adhesion molecules, inflammatory factors, and acute phase proteins. Our data also show that the protein expression levels of inflammation related factors, MMP2 and MMP9, were significantly down-regulated by tilianin through inhibition of NF-κB in VSMCs (Supplementary Figure 2).

Inflammation or ox-LDL induced migration, proliferation and phenotype conversion of VSMCs play significant roles in AS progression (Oinuma et al., 1997; Cherepanova et al., 2009; Kiyan et al., 2014; Liu et al., 2014). The abnormal proliferation and migration of VSMCs induced by ox-LDL were also proved in our present study.

Smooth muscle 22α, an actin-binding protein, is one of the biomarkers of contractile phenotype of VSMCs. Contractile phenotype of VSMC will transform into synthetic one when it suffers mechanical stretch or inflammation, and such phenotypic transformation is closely associated with proliferative ability of VSMC, which would aggravate the Atherosclerotic plaques. It was reported that the mice lacking *SM22α* gene showed significant vascular injury in the carotid artery, accompanied by increased activation, nuclear translocation and pro-inflammatory activity of NF-κB (Shu et al., 2015). Tilianin could significantly inhibit the decrease of *SM22α* expression induced by either TNF-α or ox-LDL in VSMCs, indicating that the phenotype transformation of VSMCs from the normal contractile type to the inflammatory synthetic type could be suppressed by tilianin (Figures 3C,D and Supplementary Figure 3). Therefore, the above results showed that one of the mechanisms of tilianin in amelioration of AS was through inhibition of inflammation.

Macrophages involved foam cell formation is a marker of early AS lesions in both human AS disease and AS animal models. Foam cells are defined as cells that contain a lot of lipid droplets, which can be stained by oil red O (Mallat, 2014). Aiming to define the potential mechanism by which tilianin reduced lipid content in the macrophages and explain the reason for this reduction in foam cell formation, ox-LDL induced lipid accumulation model was constructed in macrophages. The accumulation of large amounts of lipid droplets in macrophages resulted from the increase of cholesterol uptake into the cells and the decrease of cholesterol efflux from the cells. On the one hand, tilianin inhibited the expression of *SRA1* to reduce the uptake of cholesterol. On the other hand, tilianin elevated the expression of cholesterol efflux genes like *SRB1* and *ABCA1* to promote cholesterol efflux. Therefore, another possible mechanism of tilianin to ameliorate the formation of AS plaques was through the inhibition of foam cell formations.

Oxidized low density lipoprotein can damage endothelial cells and VSMCs, enhancing the inflammation of blood vessels (Wiesner et al., 2010; Maskrey et al., 2011). Our results showed that the mRNA expression levels of inflammatory cytokines and oxidative stress that led to endothelial injury were all up-regulated by ox-LDL, but tilianin had no significantly inhibitory effects on the ox-LDL induced endothelial injury. EC dysfunction is initial step of AS. Injured EC can secret adhesion molecules (IACM, etc.), chemokines (MCP-1, etc.) and various cytokines (IL-6, IL-18) to promote monocyte recruitment and adhesion to intima (Ross, 1999). To investigate whether tilianin has effects on monocyte-EC interaction, we performed monocyte transwell migration assay. Results show that tilianin can significantly inhibit monocyte recruitment caused by the supernatant from TNF-α-stimulated HUVEC (Supplementary Figure 4). However, there evidence still needs to be completely elucidated in our future researches.

## Conclusion

Advanced basic science has proved that AS involves an ongoing inflammatory response. Chinese herbal medicine tilianin has been proved to exhibit significant anti-atherosclerotic activity. However, its mechanism has not been clearly explained. In the present study, we proved that the anti-atherosclerotic mechanisms of tilianin were at least partly related to its suppression of inflammation on macrophages and VSMCs through meditating TNF-α/NF-κB pathway, inhibition of proliferation, migration and abnormal phenotype transition of VSMCs, as well as its inhibition of macrophage foam cell formation, enhancement of cholesterol efflux. Thus, our study provides some theoretical basis for the further study of tilianin on research of AS treatment.

## Author Contributions

All authors listed have made a substantial, direct and intellectual contribution to the work, and approved it for publication.

## Conflict of Interest Statement

The authors declare that the research was conducted in the absence of any commercial or financial relationships that could be construed as a potential conflict of interest.

## Abbreviations

AS: atherosclerosis
eNOS: endothelial nitric oxide synthase
GSH-PX: glutathione peroxidase
iNOS: inducible nitric oxide synthase
ICAM-1: intercellular adhesion molecule
IL-6: interleukin-6
IL-18: interleukin-18
IL-1β: interleukin-1β
MCP1: monocyte chemotactic protein 1
MMP2: matrix metalloproteinase-2
MMP9: matrix metalloproteinase-9
NF-κB: nuclear factor-kappa B
ox-LDL: oxidized low density lipoprotein
SM22α: smooth muscle 22α
SRA: class A scavenger receptor
SRB1: scavenger receptor class B type 1
SOD: superoxide dismutase
VCAM-1: vascular cell adhesion molecule.
