# Supplementary material for: Atheroprotective Mechanisms of Tilianin by Inhibiting Inflammation Through Down-Regulating NF-κB Pathway and Foam Cells Formation
Source: Front Physiol. 2019 Jul 2;10:825. doi: 10.3389/fphys.2019.00825 (PMC6614704; doi:10.3389/fphys.2019.00825)
Supplement: Supplementary file 1 [file Table_1.docx]

Supplementary Material

**Atheroprotective Mechanisms of Tilianin by Inhibiting Inflammation Through Down-Regulating NF-ΚB Pathway and Foam Cells Formation**

Wanli Shen, Gulinigaer Anwaier, Yini Cao, Guan Lian, Cong Chen, Shu Liu, Nuerbiya Tuerdi and Rong Qi*

*** Correspondence:**

Prof. Rong Qi

[ronaqi@bjmu.edu.cn](mailto:ronaqi@bjmu.edu.cn)

1. **Supplementary Method**

**1.1 Transwell Migration assay**

HUVEC cells were incubated with TNF-α and Tilianin as described in our Method section. Then, cell supernatants were obtained and centrifuged. 700 µL of medium containing above culture supernatants were added into the lower chamber. Then the membrane were inserted. 100µL monocytes incubated in DMEM without FBS were added into the upper chamber and co-cultured in cell incubator (37 °C, 5% CO2). After 24 h of co-culture, the membrane were washed with PBS for three times and incubated with 0.1% crystal violet at room temperature for 30 min. Migration of monocytes were observed in optics microscope.

1. **Supplementary Tables and Figures**
   1. **Supplementary Tables**

**Supplementary Table** **1**. Quantification of tilianin by determination of peak areas

| Number | Retention Time | Concentration | Peak Area |
| --- | --- | --- | --- |
| 1 | 10.936 | 0.07535 | 9798 |
| 2 | 14.051 | 0.7382 | 95994 |
| 3 | 14.336 | 0.1343 | 17465 |
| 4 | 14.901 | 0.09606 | 12491 |
| 5 | 15.986 | 98.68 | 12830786 |
| 6 | 16.519 | 0.2585 | 33610 |
| 7 | 17.703 | 0.01949 | 2534 |
| Total |  | 100 | 13002678 |

**Supplementary Table** **2**. Sequences of primers used for RT-PCR analysis.

Mice：

| **Genes** | **Forward (5'→ 3')** | | **Reverse (5'→ 3')** |
| --- | --- | --- | --- |
| TNF-α | | CTGTGAAGGGAATGGGTGTT | CAGGGAAGAATCTGGAAAGGTC |
| MCP-1 | | TCAATGACCCACCAGTC | AAGGAGCCATAGAGGAAC |
| SREBP-1c | | GCAGCCACCATCTAGCCTG | CAGCAGTGAGTCTGCCTTGAT |
| IL-10 | | CCCTTTGCTATGGTGTCCTT | TGGTTTCTCTTCCCAAGACC |
| IL-6 | | TAGTCCTTCCTACCCCAATTTCC | TTGGTCCTTAGCCACTCCTTC |
| NF-κB | | TCAGTGGGAATTTCCAGCCAGG | CTTTGCAGGCCCCACATAGT |
| SRA | | AGGGAGTGGATAAATCAGTGCT | TCCTCCTGTTGCTTTGCTGT |
| SRB1 | | GCCTCTGTTTCTCTCCCACC | CTGTCCGCTGAGAGAGTCCT |
| CXCL16 | | GTTGCAGTCCAAAAGCGTGT | CTGTTGCACAGCACATAGGC |
| ABCG1 | | GGTTGCGACATTTGTGGGTC | GAAGATGGTCCTCAGGTGGC |
| CD36 | | CTCGGATGGCTAGCTGATTACT | AGCACTTGCTTCTTGCCAAC |
| ABCA1 | | CGACCATGAAAGTGACACGC | GACAGCTGGCAGGACAATCT |
| β-actin | | GGCTGTATTCCCCTCCATCG | CCAGTTGGTAACAATGCCATGT |

# Rats:

| **Genes** | **Forward (5'→ 3')** | | **Reverse (5'→ 3')** |
| --- | --- | --- | --- |
| TNF-α | | AAGGGAATTGTGGCTCTGGG | GGGAACAGTCTGGGAAGCTC |
| IL-6 | | ACTTCACAAGTCGGAGGCTT | AGCATTGGAAGTTGGGGTAGG |
| NF-κB | | TGGCAGACGACGATCCTTTC | GGTATGGGCCATCTGTTGAC |
| VCAM-1 | | ACAGCTCCTCTCGGGAAATG | AGCACATGTCAGAACAACGG |
| ICAM-1 | | CGTGACCTGGACACACCTAC | TGTCCCAGCTTTCCCATGTC |
| IL-1β | | CTGTAGCATCCACGTGCTGT | AGCATTGGAAGTTGGGGTAGG |
| β-actin | | GCAGGAGTACGATGAGTCCG | ACGCAGCTCAGTAACAGTCC |

# 2.2 Supplementary Figures

**Supplementary Figure 1**. The purity of tilianin detected by HPLC.


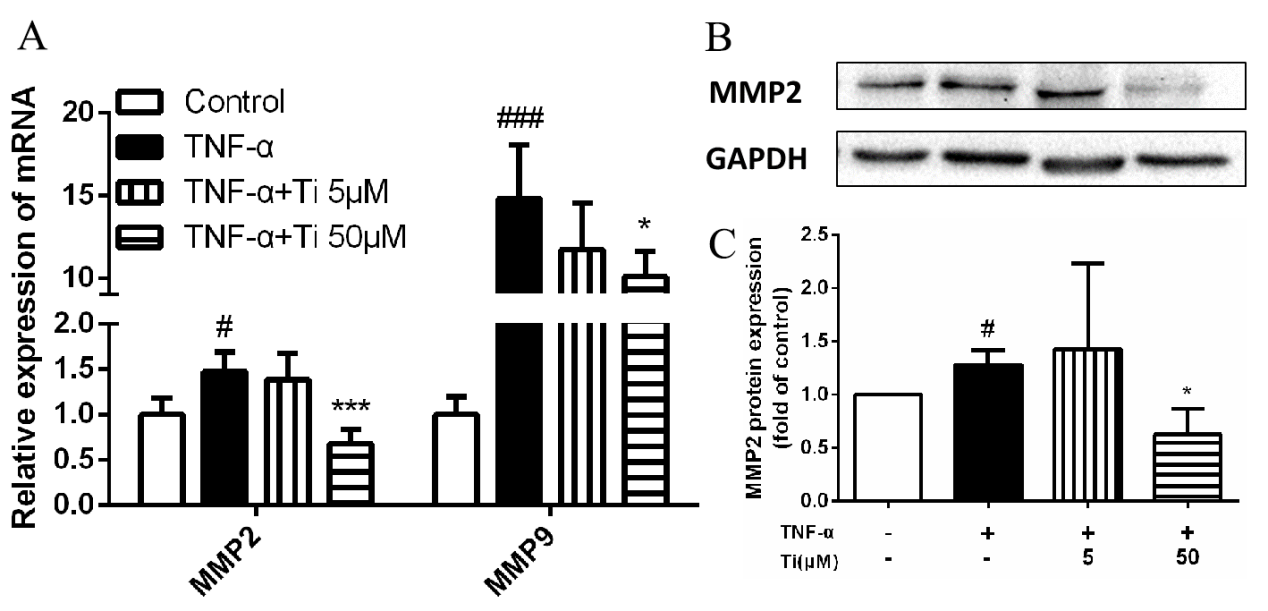


**Supplementary Figure 2**. Effects of tilianin on (A) MMP2/MMP9 mRNA expression and (B & C) MMP2 protein expression in TNF-α induced VSMCs. All data represent the mean ± SEM. ^#^P < 0.05,  ^###^ P < 0.001 *v.s* Control group, *P < 0.05, ***P < 0.0001 *v.s* TNF-α group.


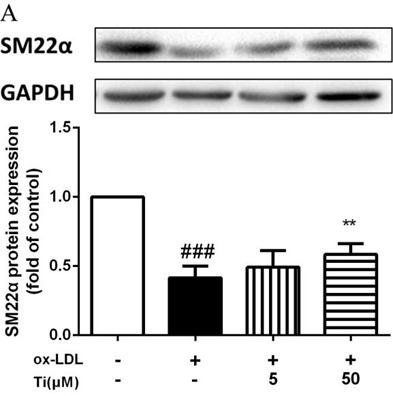


**Supplementary Figure 3**. Effects of tilianin on SM22α protein expression in ox-LDL induced VSMCs. All data represent the mean ± SEM. ^###^P < 0.001 *v.s* Control group, **P < 0.01 *v.s* TNF-α group.


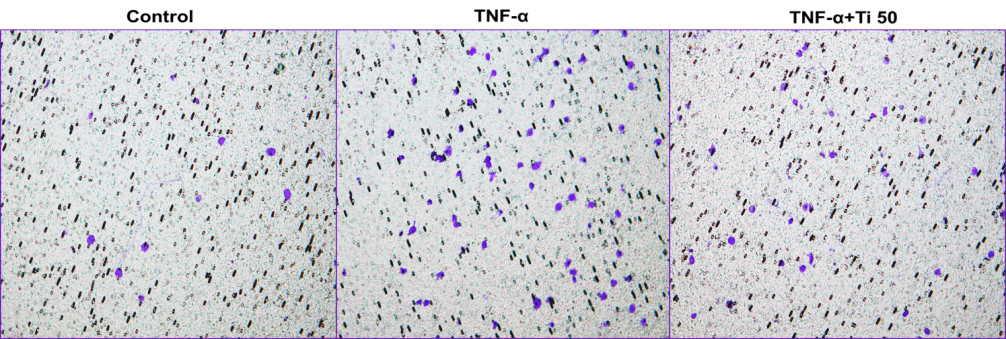




**Supplementary Figure 4**. Effects of tilianin on monocyte migration. Purple dots represent crystal violet staining positive monocytes. All data represent the mean ± SEM. ^###^ P < 0.001 *v.s* Control group, **P < 0.01 *v.s* TNF-α group.


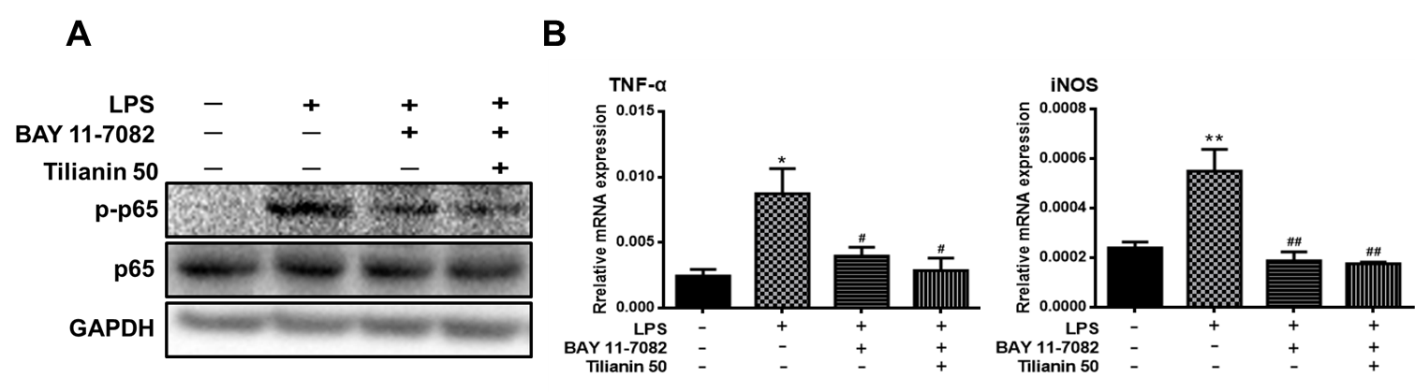


**Supplementary Figure 5**. Effects of tilianin on LPS induced inflammatory response of macrophages in the presence of inhibitors against the NF-κB. All data represent the mean ± SEM. *P < 0.05, **P < 0.001, ***P < 0.0001 *v.s* Control group; ^#^P < 0.05, ^##^P < 0.01, ^###^P < 0.001.


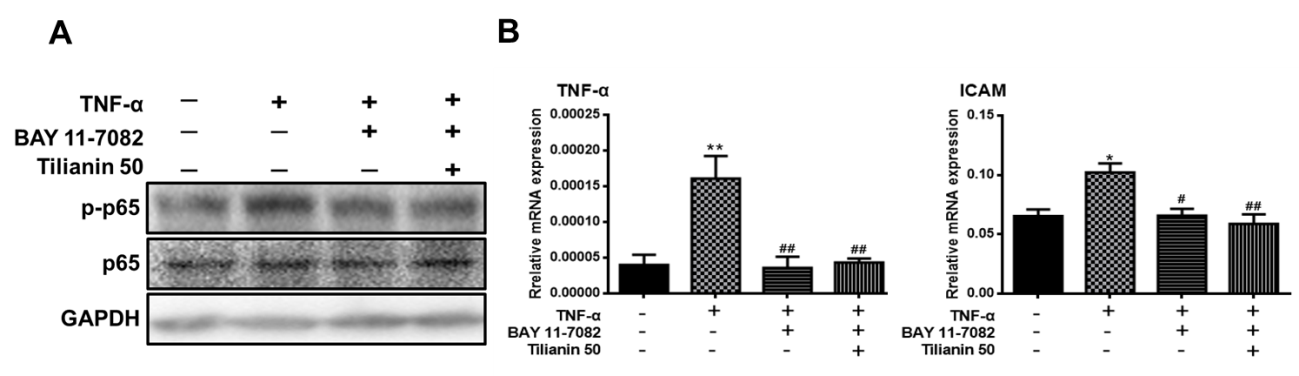


**Supplementary Figure 6**. Effects of tilianin on TNF-α induced inflammatory response of VSMC in the presence of inhibitors against the NF-κB. All data represent the mean ± SEM. *P < 0.05, **P < 0.001, ***P < 0.0001 *v.s* Control group; ^#^P < 0.05, ^##^P < 0.01, ^###^P < 0.001.
